# MiR-423-5p activated by E2F1 promotes neovascularization in diabetic retinopathy by targeting HIPK2

**DOI:** 10.1186/s13098-021-00769-7

**Published:** 2021-12-28

**Authors:** Qing Xiao, Yinu Zhao, Hongjing Sun, Jia Xu, Wenjie Li, Limo Gao

**Affiliations:** 1 Department of Ophthalmology, The Second Affiliated Hospital of Zhejiang University School of Medicine, Hangzhou, 31009 Zhejiang Province People’s Republic of China; 2 Department of Ophthalmology, The Third Xiangya Hospital, Central South University, Changsha, 410013 Hunan Province People’s Republic of China

**Keywords:** Angiogenesis, Diabetic retinopathy, E2F1, miR-423-5p, HIPK2, HIF1α/VEGF signaling

## Abstract

**Background:**

Diabetic retinopathy (DR) is a diabetic complication and the primary cause of blindness in the world. However, the treatments of DR are challenging given its complicated pathogenesis. Here, we investigated the molecular mechanisms of DR by focusing on the function of E2F1/miR-423-5p/HIPK2/HIF1α/VEGF axis.

**Methods:**

Cultured retinal endothelial cells (hRMECs, hRECs) were treated with 25 mM glucose to mimic the high glucose-induced DR in vitro. Streptozotocin (STZ) was injected into mice to induce DR in mice. qRT-PCR, western blotting, immunohistochemistry, and ELISA were employed to measure levels of E2F1, miR-423-5p, HIPK2, HIF1α, and VEGF. H&E staining was utilized to examine retinal neovascularization. CCK-8 assay, transwell assay, and vascular tube formation assay were used to assess the cell viability, migration, and angiogenesis. Dual luciferase assay was performed to validate interactions between E2F1 and miR-423-5p, miR-423-5p and HIPK2.

**Results:**

HG treatment increased the cell viability, migration, and angiogenesis accompanied by upregulation of E2F1, miR-423-5p, HIF1α, and VEGF levels, but reduction in HIPK2 expression. Knockdown of E2F1 or miR-423-5p suppressed the HG-induced increases in cell viability, migration, and angiogenesis. E2F1 transcriptionally activated miR-423-5p expression and miR-423-5p mimics blocked the effects of E2F1 knockdown on angiogenesis. Moreover, miR-423-5p directly targeted HIPK2 to disinhibit HIF1α/VEGF signaling. Knockdown of HIPK2 reversed the effects of miR-423-5p inhibitor on cell viability, migration, and angiogenesis. Knockdown of E2F1 suppressed neovascularization during DR in vivo.

**Conclusions:**

E2F1 activates miR-423-5p transcription during DR to promote angiogenesis via suppressing HIPK2 expression to disinhibit HIF1α/VEGF signaling. Strategies targeting E2F1/miR-423-5p/HIPK2 axis could be potentially used for DR treatment.

## Background

Diabetes is a very serious public health concern globally [1]. The most common complication of diabetes is diabetic retinopathy (DR), a condition that damages retina [2]. It also ranks as the leading cause of blindness worldwide [3]. DR is a microvascular disease and can be clinically divided into two stages: non-proliferative DR (NPDR) characterized by enhanced vascular permeability and capillary occlusion, and proliferative DR (PDR) featured by retinal neovascularization [4]. The development and progression of DR are tightly associated with the severeness of diabetes. Unfortunately, the treatments of DR at the present are limited as a result of its complex pathogenesis [4, 5]. Understanding the mechanisms of DR, particularly the roles of neovascularization in DR, is very critical for the development of new therapy.

E2F transcription factor 1 (E2F1) is a member of the E2F transcription factor family that is crucial downstream effector of growth factor signaling [6]. It has been shown that E2F1 plays critical roles in regulating cell cycle progression, cell death, and development [7, 8]. In addition, some studies indicated important functions of E2F1 in angiogenesis and oxidative stress [9, 10], two processes crucial for diabetes. Indeed, a recent study showed that E2F1 mediated diabetic retinal neuronal death [11]. Nevertheless, the role of E2F1 in angiogenesis during DR is not well studied. As a transcription factor, E2F1 can regulate expression of multiple microRNAs (miRNAs) [12, 13]. The downstream effector of E2F1 in retinal cells remains unknown. MiRNAs are a well-known class of endogenous non-coding RNAs that can negatively regulate gene expression via directly binding to the 3′-untranslated region (3′-UTR) of target massager RNAs (mRNAs) [14]. Emerging evidence shows that miRNAs have important roles in multiple cellular processes, including physiological processes and diseases [15]. MiR-423-5p was reported highly expressed during DR [16]. Its level was elevated in plasma of DR patients [16]. However, the detailed function of miR-423-5p in DR is incomplete and requires further characterization. Also, whether E2F1 regulates miR-423-5p expression is unknown.

Hypoxia inducible factor 1α (HIF1α) is a member of HIF transcription factor family that is induced during hypoxia and acts to activate many downstream genes including the vascular endothelial growth factor (VEGF) [17]. HIF1α/VEGF signaling has been heavily implicated in the angiogenesis during many conditions, such as joint osteoarthritis, ischemia, and cancers [18]. In DR, enhanced HIF1α/VEGF signaling pathway has been observed and is closely associated with the retinal angiogenesis [19]. Homeodomain-interacting protein kinase 2 (HIPK2) is a serine/threonine homeodomain-interacting kinase [20]. Previous studies have shown that HIPK2 inhibits tumor growth by suppressing the angiogenesis through binding to HIF1α and promoting its degradation [21]. However, whether HIPK2/HIF1α interaction is involved in DR remains largely unknown.

Our preliminary bioinformatic analysis (http://www.targetscan.org/vert_71/; Target score: 89) implied that miR-423-5p could target HIPK2 and we hypothesized that E2F1/miR-423-5p/HIPK2/HIF1α axis may regulate the angiogenesis to participate in DR. In this paper, our study shows that E2F1 activates miR-423-5p transcription during DR. miR-423-5p directly targets HIPK2 and its activation during DR promotes neovascularization via suppressing HIPK2 to disinhibit HIF1α/VEGF signaling. These results provide insights into the molecular mechanisms of DR progression, as well as avenues for the development of efficient therapy.

## Methods

### Cell culture

Human retinal microvascular endothelial cells (hRMECs) and human primary retinal endothelial cells (hRECs) which are the classical cells to study the neovascularization in DR were purchased from the Cell Bank of Chinese Academy (Shanghai, China). The medium used for cell culture was composed of human endothelial cell medium, 10% fetal bovine serum (FBS, Invitrogen, USA) and 1% penicillin-streptomycin (Gibco, USA). The cells were seeded onto the plate that was pre-coated with 1% gelatin (Sigma-Aldrich, MO, USA) and maintained in the CO_2_ incubator at 37 °C. To mimic the neovascularization during DR in vitro, cells were cultured in medium with high glucose concentration (HG, 25 mM). For normal glucose (NG) condition, the glucose concentration was 5 mM. Cells were cultured in these mediums for 48 h before subsequent experiments.

### Plasmids and cell transfection

E2F1 full length was cloned into the overexpression plasmid (pcDNA-3.1, oe-E2F1). MiR-423-5p mimics and inhibitor, sh-E2F1, sh-HIPK2, and control sh-NC were synthesized from Genepharma (Shanghai, China). Lipofectamine 3000 (Invitrogen, Missouri, USA) was utilized as the reagent for cell transfection. In brief, cells were cultured to ~ 70% confluence, and then construct was added together with Lipofectamine 3000 at a ratio of 1:1. Cells were harvested for further analysis 48 h after transfection.

### Dual luciferase assay

HIPK2 fragment of wild-type (WT) sequence or mutant (MUT) sequence with the binding sites with miR-423-5p was inserted into the pmirGLO vector (Ribobio, Guangzhou, China) to make the luciferase reporter plasmids (HIPK2-WT or HIPK2-MUT). The construct (HIPK2-WT/MUT) was transfected into cells together with miR-423-5p mimics or mimics NC. 48 h after transfection, transfected cells were harvested and luciferase activity of each condition was measured by using the Dual Luciferase Reporter Assay System (Promega). Regarding miR-423-5p promoter luciferase assay, the WT sequence or MUT sequence with binding sites of E2F1 in miR-423-5p promoter region (miR-423-5p-WT, miR-423-5p-MUT) were amplified and subsequently cloned into the pGL3-Basic vector (Promega). Cells were seeded into the wells of 24-well plate at the density of 1 × 10^5^ cells/well. After cells were attached to the plate, pGL3-miR-423-5p promoter construct (WT or MUT) was transfected into cells together with oe-E2F1, or its corresponding negative control (oe-NC).

### Enzyme-linked immunosorbent assay (ELISA)

Secreted VEGF level was quantified by using the commercial ELISA kit (ab100662, Abcam, USA) as the manufacturer’s protocol described. Briefly, culture medium was collected and incubated with primary anti-VEGF antibody in the 96-well plate together with standard samples for 1–1.5 h at room temperature. Horseradish peroxidase-conjugated secondary antibody was added to incubate with samples for additional 1 h. The plate was washed with PBS and analyzed with a spectrophotometer.

### Cell counting kit-8 (CCK-8)

Cell proliferation was measured using the commercial CCK-8 kit (ab228554, Abcam) as the protocol described. Cells were seeded in the 96-well plates at a density of 2 × 10^3^ cells/well and cultured in the incubator for 0, 24, 48 and 72 h. 10 µL CCK-8 solution was added to incubate with cells for 2 h at 37 °C. The absorbance at 450 nm was analyzed with the standard microplate reader.

### Transwell assay

Transfected hRMECs or hRECs were seeded in the culture medium with no serum on top of the filter membrane (8 μm pore) that was precoated with Matrigel (Corning, USA). Full culture medium that contains 10% FBS was put in the lower chamber. 24 h after the upper filter was discarded. Cells growing on the lower dish were cells migrated there from the filter. They were fixed in 4% paraformaldehyde first for 10–15 min at room temperature, and then 0.1% crystal violet was added to stain the cells followed by imaging.

### Vascular tube formation assay

24-well plates were pre-coated at 4℃ with Matrigel solution (BD, Biosciences) and incubated at 37℃ for 1 h to allow for polymerization of the Matrigel. Transfected hRMEC or hREC cells were seeded in the Matrigel-coated wells under NG or HG conditions. After incubation at 37℃ for 6 h, images were taken by using an inverted microscope. Tube formation was quantified by ImageJ software.

### RNA extraction and qRT-PCR

Trizol (Invitrogen, USA) was employed to extract total RNAs from cultured cells and tissues as the manufacturer’s instruction described. For miRNA analysis, total RNAs were isolated with the miRNeasy Advanced Mini Kit (QIAGEN, Hilden, Germany). DNaseI was included into the lysis buffer to avoid the contamination of DNA. Commercial cDNA synthesis Kit (Thermo Fisher Scientific, USA) was utilized to generate cDNAs through reverse transcription. SYBR Green Master Mix (Invitrogen, USA) was used for the quantitative PCR. Relative expression level of miR-423-5p was measured using specific miR-423-5p miScript Primer Assay (SABiosciences, MD, USA). U6 was served as an internal standard for miR-423-5p and β-actin was served as an internal standard for E2F1 and HIPK2. The relative expression level was calculated by 2^−ΔΔCt^ method. The primers listed as follows were from Genepharma (Shanghai, China):

miR-423-5p forward primer (FP) : 5′-TGAGGGGCAGAGAGCGA-3′;

miR-423-5p reverse primer (RP) :

5′-GTCGTATCCAGTGCAGGGTCCGAGGTATTCGCACTGGATACGACAAAGTC-3′;

U6 FP 5′- TGGCGGGTGTATTAAACCAC-3′,

U6 RP: 5′-TTCACGAATTTGCGTGTCATC-3′.

E2F1 FP: 5′-GGATTTCACACCTTTTCCTGGAT-3′;

E2F1 RP: 5′-CCTGGAAACTGACCATCAGTACCT-3′;

HIPK2 FP: 5′-CCACATGTCAATTGCCTCAC-3′;

HIPK2 RP: 5′-AGGTCATTGACTTTGGTTCAG-3′;

β-actin FP: 5′-CCCTGGAGAAGAGCTACGAG-3′;

β-actin RP: 5′-CGTACAGGTCTTTGCGGATG-3′.

### Western blotting

Proteins from cultured cells were extracted by utilizing the RIPA lysis buffer (Abcam, USA) according to standard protocol. DC Protein Assay Kit (Bio-Rad, USA) was utilized to quantify the protein concentration. Equal protein from each sample was loaded into SDS-polyacrylamide gels and separated through electrophoresis. Later proteins in the gels were transferred to PVDF membranes (Sigma-Aldrich, USA). 3% BSA was used to block the membranes for 30–60 min at room temperature and then specific primary antibodies were added to incubate at 4 °C overnight. The antibodies were discarded and TBST was utilized to wash the membranes three times before incubation with specific goat anti-rabbit (cat. no. 7074) or goat anti-mouse (cat. no. 7076) horseradish peroxidase-conjugated secondary antibodies (1:3000; Cell Signaling Technology, USA) for 1–2 h at room temperature. Protein band intensities were detected by using the ECL Kit (Bio-Rad). Primary antibodies used in the study were: Anti-E2F1 (1:1500; cat. no. 3742, Cell Signaling Technology, USA); Anti-HIPK2 (1:1500; cat. no. ab221980, Abcam, USA); Anti-HIF1α (1:2500; cat. no. ab179483, Abcam, USA); Anti-β-actin (1: 5000; cat. no. ab8226, Abcam, USA).

### Mouse model of diabetic retinopathy

All animal experiments have been reviewed and approved by Institutional Ethics Review Committee of the third Xiangya Hospital of Central South University (Hunan, China). 40 male C57BL/6 J mice (8 weeks) were purchased from Shanghai SLAC Laboratory Animal Center (Shanghai, China). The mice were randomly divided into sham, DR, DR+sh-NC, DR+sh-E2F1 groups (n = 10 per group). To induce DR, mice were fasted overnight and then intraperitoneally injected with streptozotocin (STZ, 60 mg/kg in citrate buffer, Sigma-Aldrich) once a day for 5 consecutive days as previously described [22]. The blood glucose levels were measured once a week and the mice with blood glucose levels > 300 mg/dL were considered diabetes. Sham-mice received citrate buffer injection. To knockdown E2F1 expression in vivo, sh-E2F1 sequence was cloned into the pAAV2-CMV-GFP vector to generate adeno-associated viruses (AAVs). AAVs carrying sh-E2F1 or sh-NC were injected into the vitreous body of the mice by using the Nanoject II microinjector (Drummond Scientific Company, USA) under anesthesia. The animals were injected with AAVs 4 weeks after STZ injection. Animals were sacrificed 4 months after AAVs injection for further experiments.

### Hematoxylin and Eosin (H&E) staining

Retina tissues from each group of mice were immersed in 4% paraformaldehyde buffer for overnight at 4 °C and then washed with PBS and embedded in paraffin. Embedded tissues were cut into 5 μm thick slices and stained with hematoxylin and eosin (H&E) as manufacturer’s instruction described.

### Immunohistochemistry (IHC)

Embedded sections were mounted on glass slices, dried overnight at 37 °C, and deparaffinized in xylene and then rehydrated through a graded concentration of alcohol. 3% hydrogen peroxide was used to quench the sections followed by blocking with 5% bovine serum albumin (BSA) for 1 h. The sections were incubated with primary antibody (anti-VEGF, cat. no. PA1-21796, Thermo Fisher Scientific) at 4 °C overnight. After PBS washes, the sections were incubated with secondary antibody (goat anti-rabbit IgG (H + L), cat. no. 65-6120, Thermo Fisher Scientific) for 1 h at room temperature. All slices were then incubated with substrates for Envision system-HRP using the standard kit (Abcam, UK) as the manufacturer’s protocol described. Images were taken using a light microscope.

### Statistical analysis

All experiments were carried out with at least three biological replicates and the data were analyzed in GraphPad Prism 7. Statistical details were calculated by Student’s *t* test (two groups) or one-way analysis of variance (ANOVA) followed by Tukey’s post hoc test (multiple groups). The difference was considered significant if P < 0.05. All experimental data were presented as mean ± standard deviation (SD).

## Results

### HG increased E2F1 and miR-423-5p but diminished HIPK2 in hRMEC and hREC cells

**Fig. 1:**
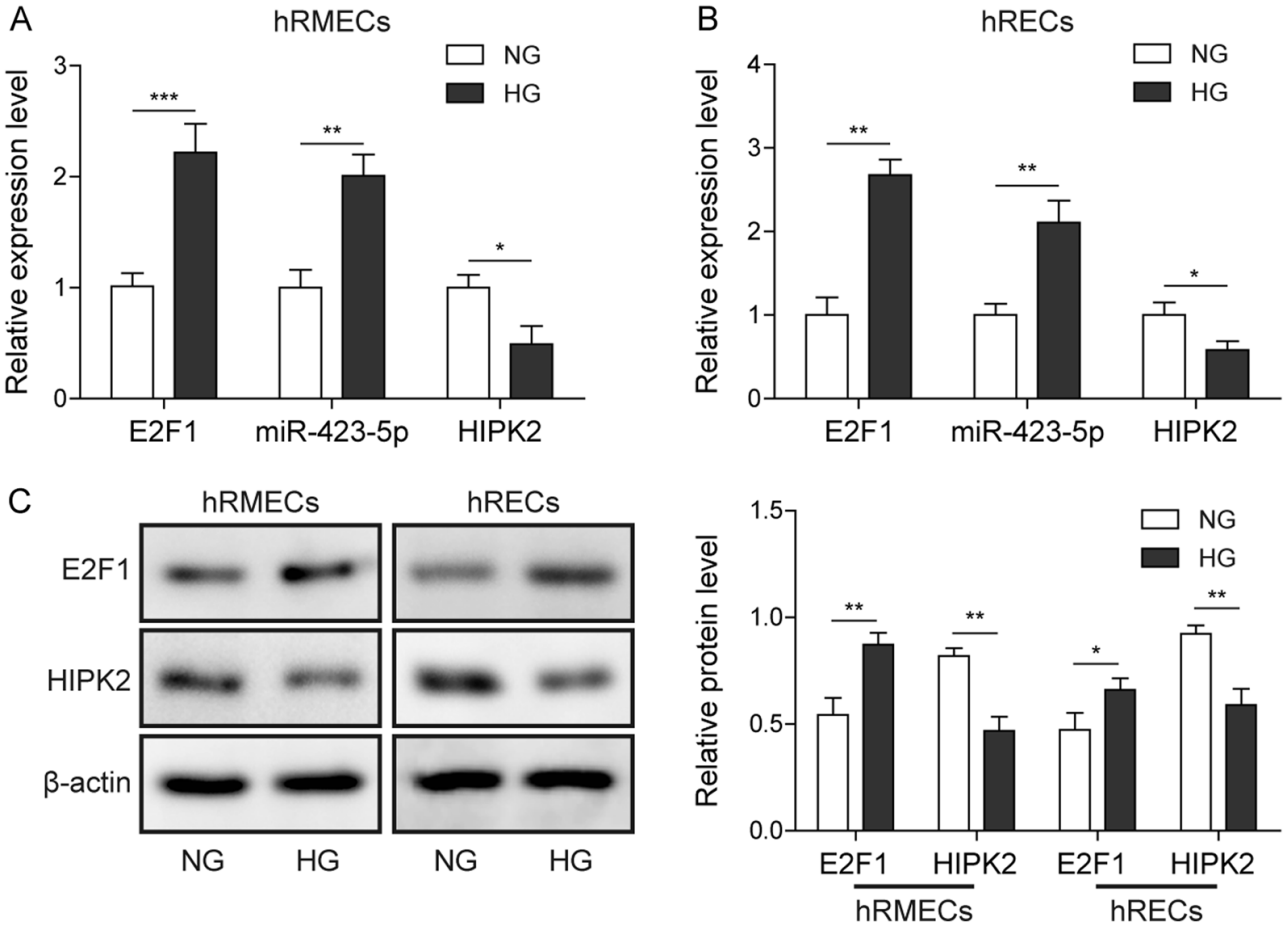
HG increased E2F1 and miR-423-5p but diminished HIPK2 expression in hRMEC and hREC cells. **A**, **B** HG increased levels of E2F1 mRNA, miR-423-5p, but decreased HIPK2 mRNA in hRMECs (**A**) and hRECs (**B**) compared to NG condition. **C** HG upregulated E2F1 protein level but downregulated HIPK2 protein level in hRMECs and hRECs compared to NG condition. All results were presented as the mean ± SD (n = 3). *P < 0.05, **P < 0.01, and ***P < 0.001. HG, high glucose; NG, normal glucose; E2F1, E2F transcription factor 1; HIPK2, homeodomain-interacting protein kinase 2

To study the functions of E2F1/miR-423-5p/HIPK2 axis in DR, we employed the cell model of DR by challenging hRMEC and hREC cells with HG condition and measured their levels. Compared to cells cultured in NG condition, E2F1 mRNA and miR-423-5p levels were greatly up-regulated in cells grown in HG condition while HIPK2 mRNA was diminished (Fig. 1A and B). Consistently, western blotting results indicated that E2F1 protein level was increased as well following HG treatment while HIPK2 protein level was decreased (Fig. 1C). These data show that HG induces robust changes of E2F1, miR-423-5p and HIPK2, implying that they might be involved in DR.

### Knockdown of miR-423-5p suppressed angiogenesis in hRMEC and hREC cells with HG condition

**Fig. 2:**
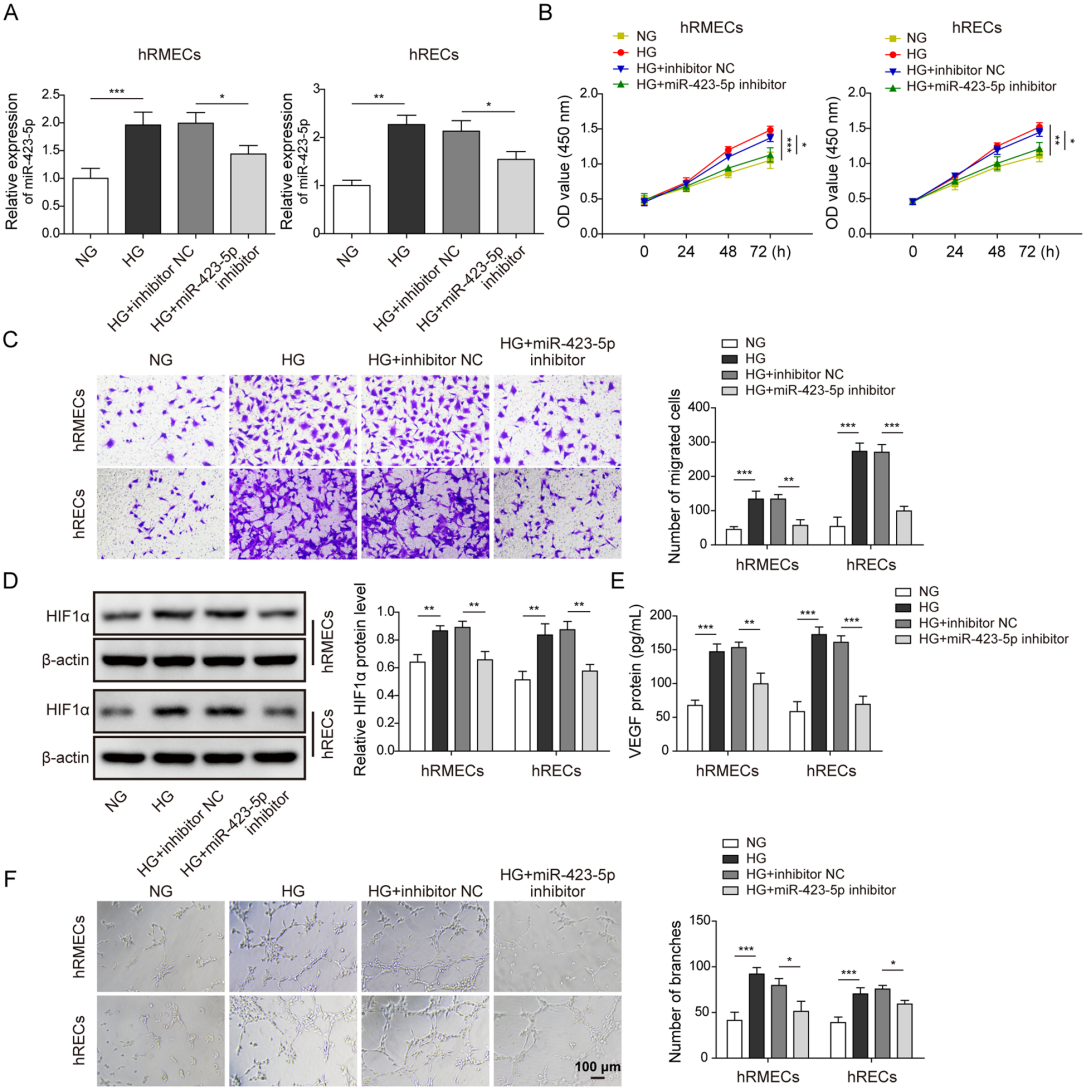
Knockdown of miR-423-5p suppressed angiogenesis in hRMEC and hREC cells with HG condition. **A** HG increased miR-423-5p level and transfection with miR-423-5p inhibitor suppressed that increase in miR-423-5p expression. **B** CCK-8 results showed HG treatment increased cell viability of hRMEC and hREC cells while miR-423-5p inhibitor suppressed the HG-induced increase in cell viability. **C** Transwell assay results indicated that HG enhanced migration of cells while transfection with miR-423-5p inhibitor blocked the enhancement caused by HG. **D** HG increased HIF1α protein level compared to NG while transfection of miR-423-5p inhibitor suppressed that increase in HIF1α protein. **E** HG increased secreted VEGF level compared to NG while transfection of miR-423-5p inhibitor suppressed that increase in secreted VEGF. **F** Vascular tube formation assay showed HG facilitated angiogenesis compared to NG while transfection of miR-423-5p inhibitor suppressed the facilitation caused by HG. All results were presented as the mean ± SD (n = 3). *P < 0.05, **P < 0.01, and ***P < 0.001. HG, high glucose; NG, normal glucose; HIF1α, hypoxia inducible factor 1α; VEGF, vascular endothelial growth factor; NC, negative control

To further study the role of miR-423-5p, we manipulated its expression level and examined the ensuing effects on angiogenesis. As expected, miR-423-5p inhibitor reversed the HG-induced up-regulation of miR-423-5p in hREMCs and hRECs (Fig. 2A). CCK-8 assay results showed that HG treatment significantly enhanced the proliferation of cells while miR-423-5p inhibitor reversed the enhancement (Fig. 2B). Similarly, as shown in Fig. 2C, transwell assay results indicated that HG increased the migration ability of hREMCs and hRECs and miR-423-5p inhibitor suppressed the effect of HG. At the molecular level, we observed that HIF1α protein and secreted VEGF levels in the medium were greatly elevated following HG exposure (Fig. 2D and E). Again miR-423-5p inhibitor brought those levels back to baseline (Fig. 2D and E). We measured the angiogenesis by the vascular tube formation assay and found that HG treatment remarkably promoted angiogenesis of hRMEC and hREC cells while miR-423-5p inhibitor reversed the effect of HG (Fig. 2F). Taken together, these results demonstrate that knockdown of miR-423-5p restrains the effects of HG on proliferation, migration, and angiogenesis abilities of hREMCs and hRECs.

### E2F1 facilitated miR-423-5p transcription

**Fig. 3:**
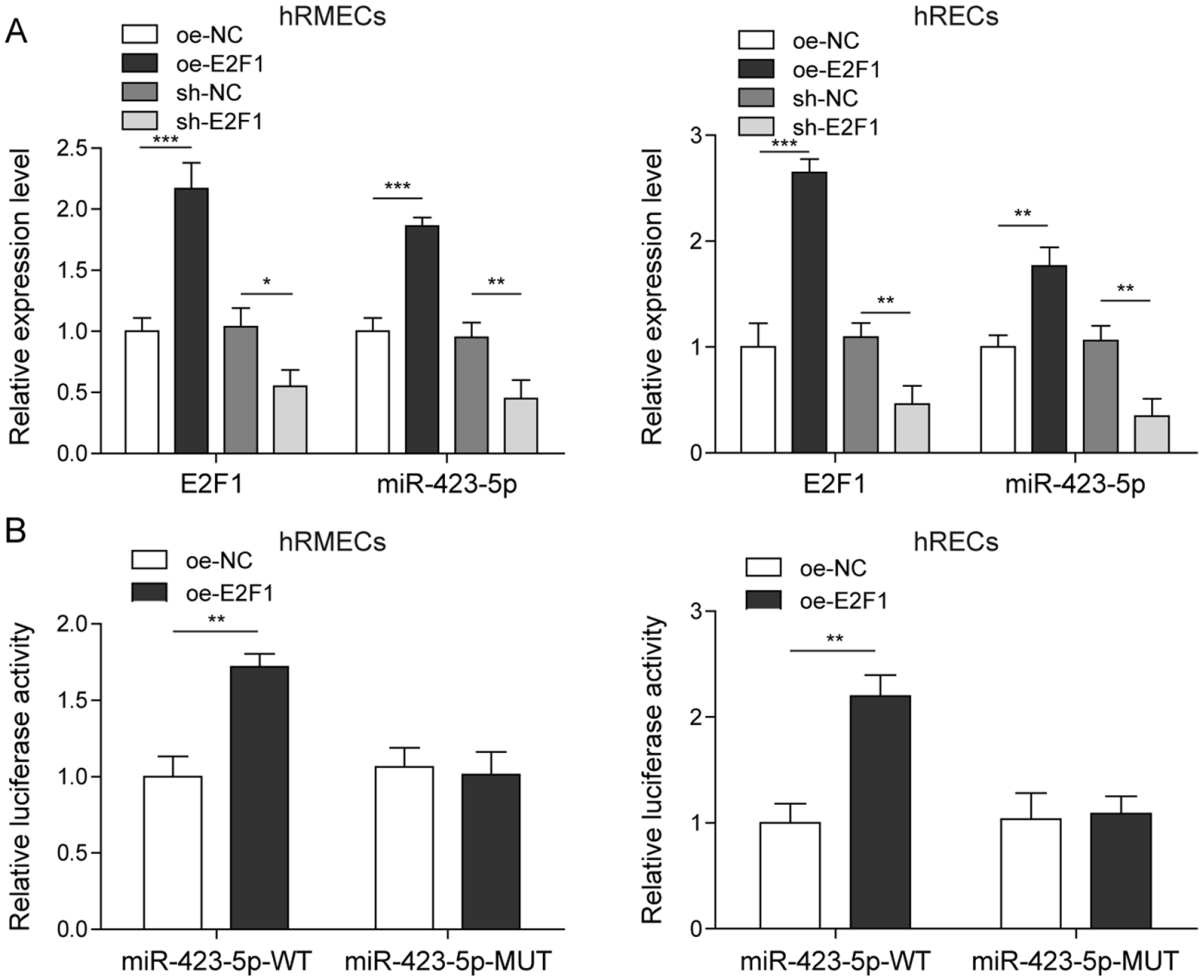
E2F1 facilitated miR-423-5p transcription. **A** Transfection of oe-E2F1 upregulated E2F1 mRNA and miR-423-5p levels while sh-E2F1 decreased both levels. **B** Transfection of oe-E2F1 increased the luciferase activity of miR-423-5p-WT but did not change the activity of miR-423-5p-MUT. All results were presented as the mean ± SD (n = 3). *P < 0.05, **P < 0.01, and ***P < 0.001. E2F1, E2F transcription factor 1; WT, wild-type; MUT, mutant; oe, overexpression

E2F1 is a transcription factor that regulates multiple gene expression [6] and thus we next examined whether E2F1 modulated miR-423-5p expression. First, we found that overexpression of E2F1 increased miR-423-5p level while knockdown of E2F1 diminished miR-423-5p expression (Fig. 3A), suggesting that E2F1 positively regulates miR-423-5p expression. Next, we employed dual luciferase assay to directly study the interaction. Transfection of cells with oe-E2F1 enhanced the luciferase activity of miR-423-5p-WT but not miR-423-5p-MUT wherein the predicted binding sites were mutated (Fig. 3B). Therefore, we conclude that E2F1 directly binds to miR-423-5p and facilitates its expression.

### Knockdown of E2F1 inhibited angiogenesis via repressing miR-423-5p expression

**Fig. 4:**
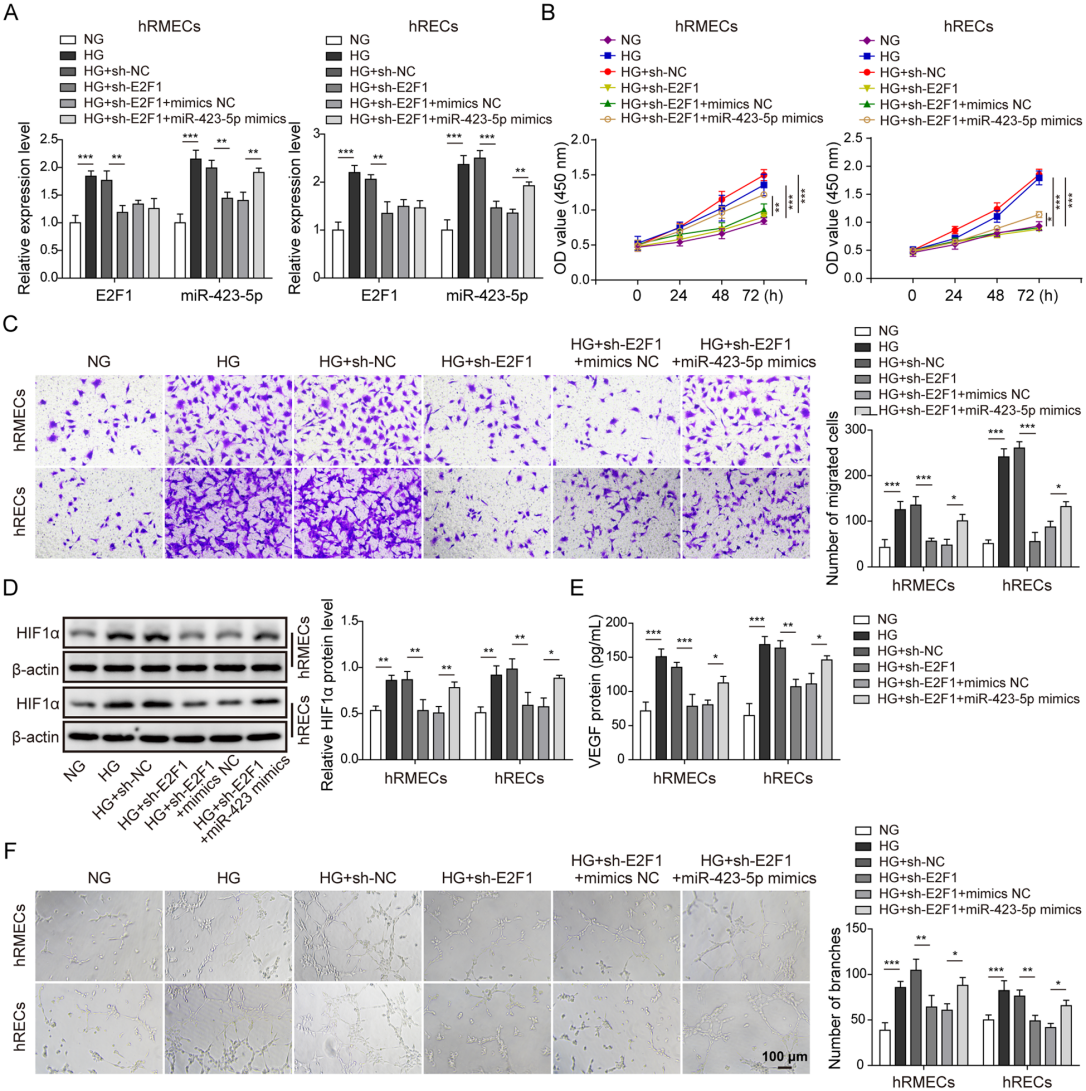
Knockdown of E2F1 inhibited angiogenesis via repressing miR-423-5p expression. **A** sh-E2F1 suppressed HG-induced increases in E2F1 mRNA and miR-423-5p levels. miR-423-5p mimics upregulated miR-423-5p level again in sh-E2F1-transfected cells following HG. **B** CCK-8 assay showed sh-E2F1 suppressed HG-induced increase in cell viability. miR-423-5p mimics increased the viability of cells transfected with sh-E2F1 after HG. **C** Transwell assay indicated that sh-E2F1 inhibited HG-induced increase in migration while miR-423-5p mimics enhanced the migration of cells transfected with sh-E2F1 following HG. **D** sh-E2F1 suppressed HG-induced in HIF1α protein level while miR-423-5p mimics increased HIF1α again in cells transfected with sh-E2F1 grown in HG. **E** sh-E2F1 suppressed HG-induced VEGF level while miR-423-5p mimics increased the level of secreted VEGF again in cells transfected with sh-E2F1 grown in HG. **F** Vascular tube formation assay showed that sh-E2F1 suppressed HG-induced angiogenesis while miR-423-5p mimics promoted angiogenesis in sh-E2F1-transfected cells grown in HG. All results were presented as the mean ± SD (n = 3). *P < 0.05, **P < 0.01, and ***P < 0.001. HG, high glucose; NG, normal glucose; E2F1, E2F transcription factor 1; HIF1α, hypoxia inducible factor 1α; VEGF, vascular endothelial growth factor; NC, negative control

Next, we sought to investigate the function of the link between E2F1 and miR-423-5p in angiogenesis. Consistent with aforementioned results, HG exposure increased E2F1 and miR-423-5p levels (Fig. 4A). Transfection of cells with sh-E2F1 suppressed the increases in E2F1 and miR-423-5p caused by HG, while miR-423-5p mimics raised miR-423-5p level again but not E2F1 (Fig. 4A). Using CCK-8 assay, we found that knockdown of E2F1 suppressed HG-induced enhancement of cell viability (Fig. 4B). However, co-transfection with miR-423-5p mimics with sh-E2F1 accelerated the proliferation again (Fig. 4B). Similarly, the increased migration ability of hRMECs and hRECs induced by HG was blocked by sh-E2F1 while co-overexpression of miR-423-5p mimics reversed the effect of sh-E2F1 (Fig. 4C). Molecularly, knockdown of E2F1 suppressed the increases of HIF1α and VEGF upon HG treatment, and miR-423-5p mimics blocked the suppression (Fig. 4D and E). Regarding the angiogenesis process, sh-E2F1 diminished the neovascularization induced by HG while miR-423-5p co-expression reversed the effect (Fig. 4F). Altogether, we provide evidence that knockdown of E2F1 represses HG-induced angiogenesis through down-regulating miR-423-5p level.

### MiR-423-5p targeted and negatively regulated HIPK2

**Fig. 5:**
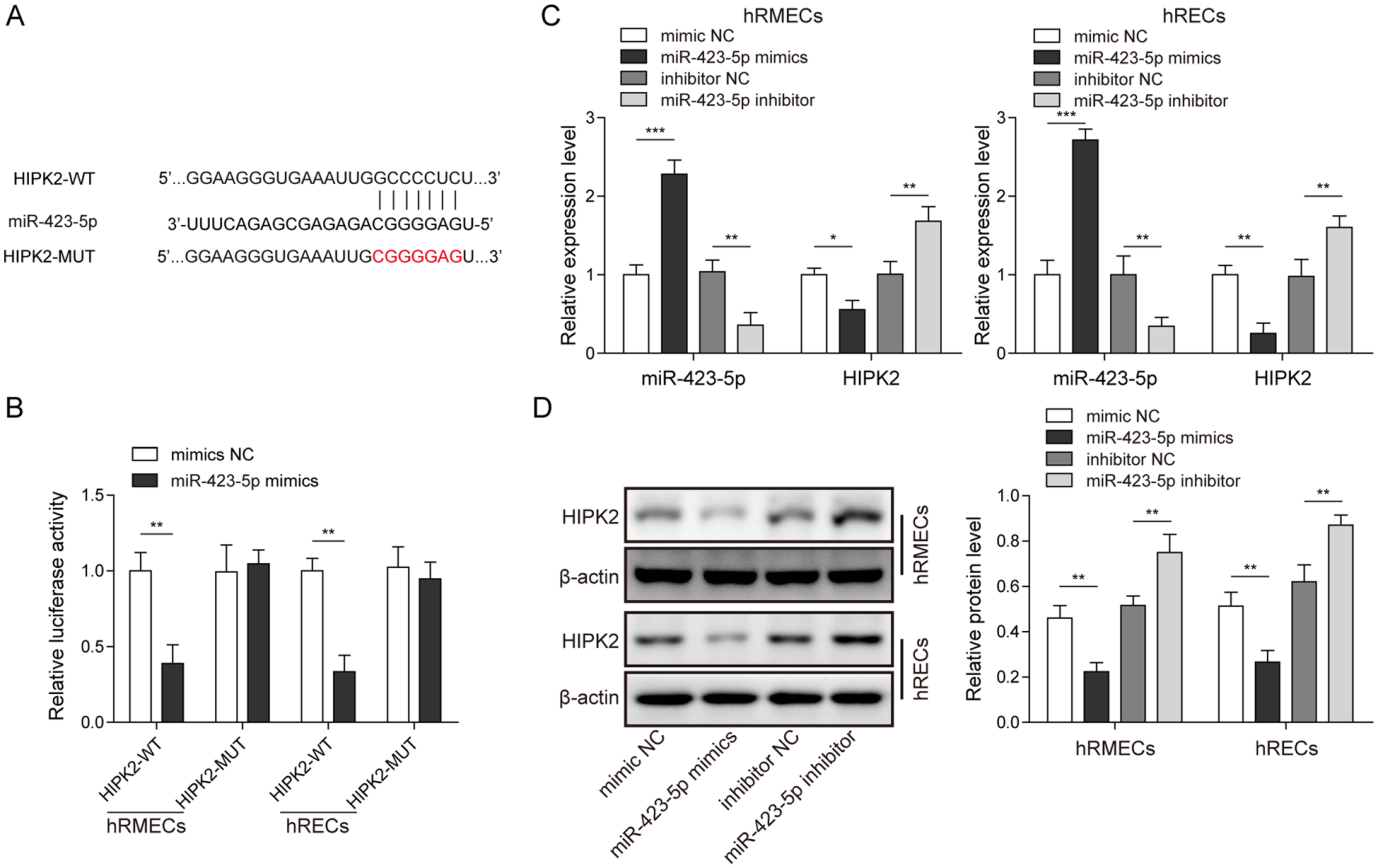
MiR-423-5p targeted and negatively regulated HIPK2 expression. **A** Complementary binding sites between miR-423-5p and HIPK2 mRNA. **B** miR-423-5p mimics diminished luciferase activity of HIPK2-WT but did not change the activity of HIPK2-MUT. **C** miR-423-5p mimics increased miR-423-5p level but reduced HIPK2 mRNA level in the cells while miR-423-5p inhibitor had opposite effects. **D** miR-423-5p mimics diminished HIPK2 protein level while miR-423-5p inhibitor increased its level. All results were presented as the mean ± SD (n = 3). *P < 0.05, **P < 0.01, and ***P < 0.001. WT, wild-type; MUT, mutant; NC, negative control; HIPK2, homeodomain-interacting protein kinase 2

MiRNAs exert functions by targeting downstream mRNAs [15]. To understand the mechanisms underlying the function of miR-423-5p, we explored the possible targets of miR-423-5p. Through bioinformatic analysis (Targetscan), we identified some complementary binding sites between miR-423-5p and HIPK2 (Fig. 5A). To validate the interaction, we employed dual luciferase activity assay. We observed that ectopic expression of miR-423-5p significantly decreased the luciferase activity of HIPK2-WT but had not effects on the activity of HIPK2-MUT in which the predicted binding sites with miR-423-5p were mutated (Fig. 5B). Further, we found that overexpression of miR-423-5p through miR-423-5p mimics greatly diminished both mRNA and protein levels of HIPK2 while downregulation of miR-423-5p level via miR-423-5p inhibitor enhanced HIPK2 expression levels (Fig. 5C and D), suggesting that miR-423-5p negatively regulates HIPK2 expression. Taken together, these results indicate that miR-423-5p directly targets and negatively regulates HIPK2.

### MiR-423-5p regulated angiogenesis via targeting HIPK2 to modulate HIF1α/VEGF signaling

**Fig. 6:**
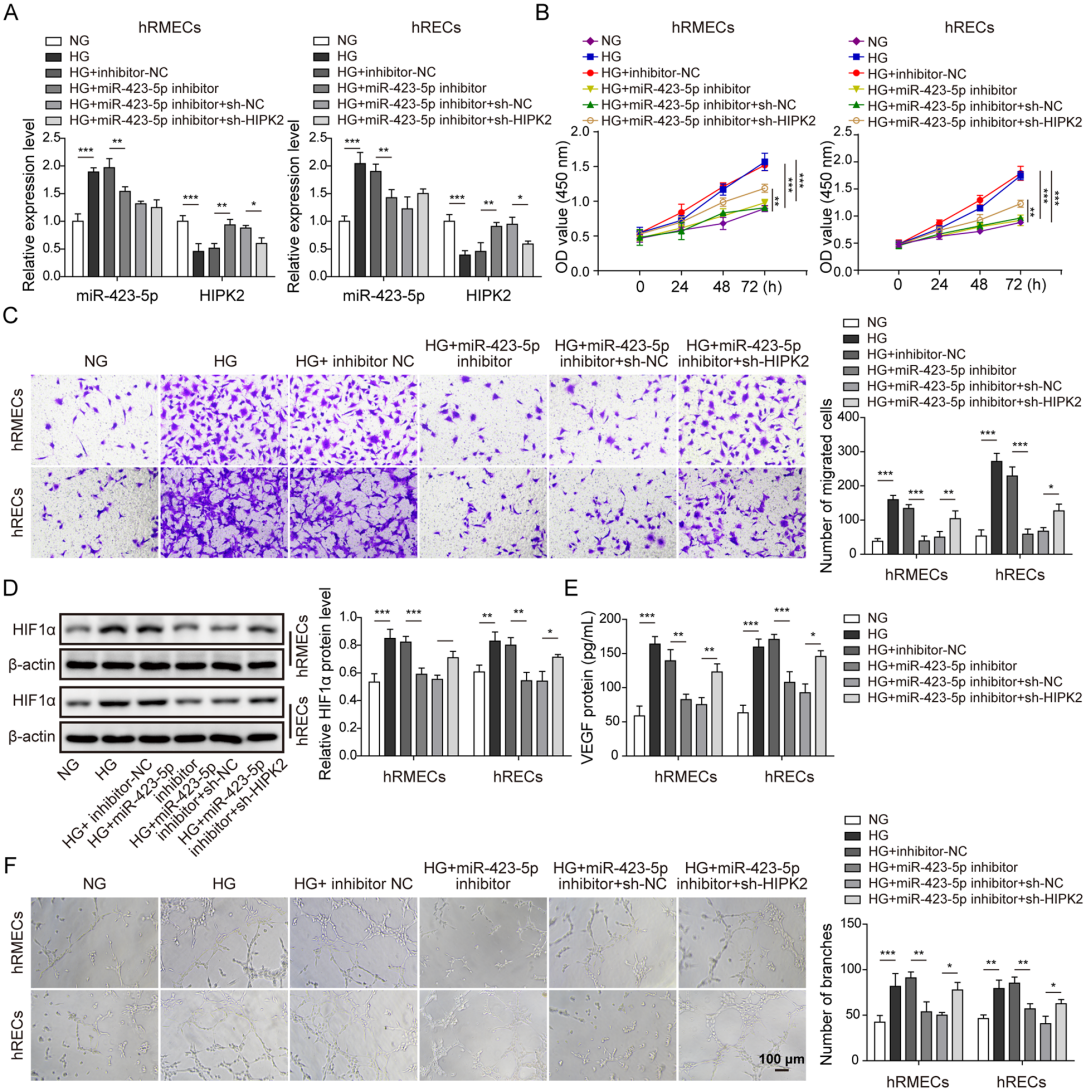
MiR-423-5p regulated angiogenesis via targeting HIPK2 to modulate HIF1α/VEGF signaling. **A** miR-423-5p inhibitor suppressed HG-induced increase in miR-423-5p level and reduction in HIPK2 mRNA level. sh-HIPK2 downregulated HIPK2 mRNA level in miR-423-5p inhibitor-transfected cells grown in HG. **B** CCK-8 assay showed miR-423-5p inhibitor suppressed HG-induced increase in cell viability. sh-HIPK2 increased the viability of cells transfected with miR-423-5p inhibitor after HG. **C** Transwell assay indicated that miR-423-5p inhibitor inhibited HG-induced increase in migration while sh-HIPK2 enhanced the migration of cells transfected with miR-423-5p inhibitor following HG. **D** miR-423-5p inhibitor suppressed HG-induced in HIF1α protein level while sh-HIPK2 increased HIF1α again in cells transfected with miR-423-5p inhibitor grown in HG. **E** miR-423-5p inhibitor suppressed HG-induced VEGF level while sh-HIPK2 increased the level of secreted VEGF again in cells transfected with miR-423-5p inhibitor grown in HG. **F** Vascular tube formation assay showed that miR-423-5p inhibitor suppressed HG-induced angiogenesis while sh-HIPK2 promoted angiogenesis in miR-423-5p inhibitor-transfected cells grown in HG. All results were presented as the mean ± SD (n = 3). *P < 0.05, **P < 0.01, and ***P < 0.001. HG, high glucose; NG, normal glucose; HIPK2, homeodomain-interacting protein kinase 2; HIF1α, hypoxia inducible factor 1α; VEGF, vascular endothelial growth factor; NC, negative control

In the end, we evaluated the role of miR-423-5p/HIPK2 interaction in angiogenesis. As expected, HG treatment up-regulated miR-423-5p level but decreased HIPK2 expression. Transfection of cells with miR-423-5p inhibitor brought miR-423-5p level back to baseline, as well as recovered HIPK2 level, while co-transfection of sh-HIPK2 with miR-423-5p inhibitor diminished HIPK2 expression again without affecting miR-423-5p level (Fig. 6A). With CCK-8 assay, we found miR-423-5p inhibitor suppressed the increase in cell viability induced by HG. However, this suppression by miR-423-5p inhibitor was reversed by co-transfection of sh-HIPK2 (Fig. 6B). Similarly, using transwell assay, we observed that miR-423-5p inhibitor significantly decreased the migration of transfected cells while knockdown HIPK2 via sh-HIPK2 restored the migration ability (Fig. 6C). We examined the molecular mechanisms by measuring the levels of HIF1α and VEGF. HG treatment upregulated the protein levels of HIF1α and VEGF while miR-423-5p inhibitor restrained those up-regulations. Knockdown HIPK2 in miR-423-5p inhibitor transfected cells partially blocked the effects of miR-423-5p inhibitor, raising the protein levels of HIF1α and VEGF again (Fig. 6D and E). Lastly, we measured the angiogenesis ability of cells through the vascular tube formation assay. HG greatly enhanced the angiogenesis ability of hRMECs and hRECs while miR-423-5p inhibitor suppressed that enhancement. Nevertheless, knockdown HIPK2 through sh-HIPK2 reversed the effects of miR-423-5p inhibitor by promoting the angiogenesis ability of transfected cells (Fig. 6F). Altogether, our results show that miR-423-5p regulates the viability, migration, and angiogenesis of retinal endothelial cells via targeting HIPK2.

### Knockdown of E2F1 suppressed angiogenesis during D***R in vivo***

**Fig. 7:**
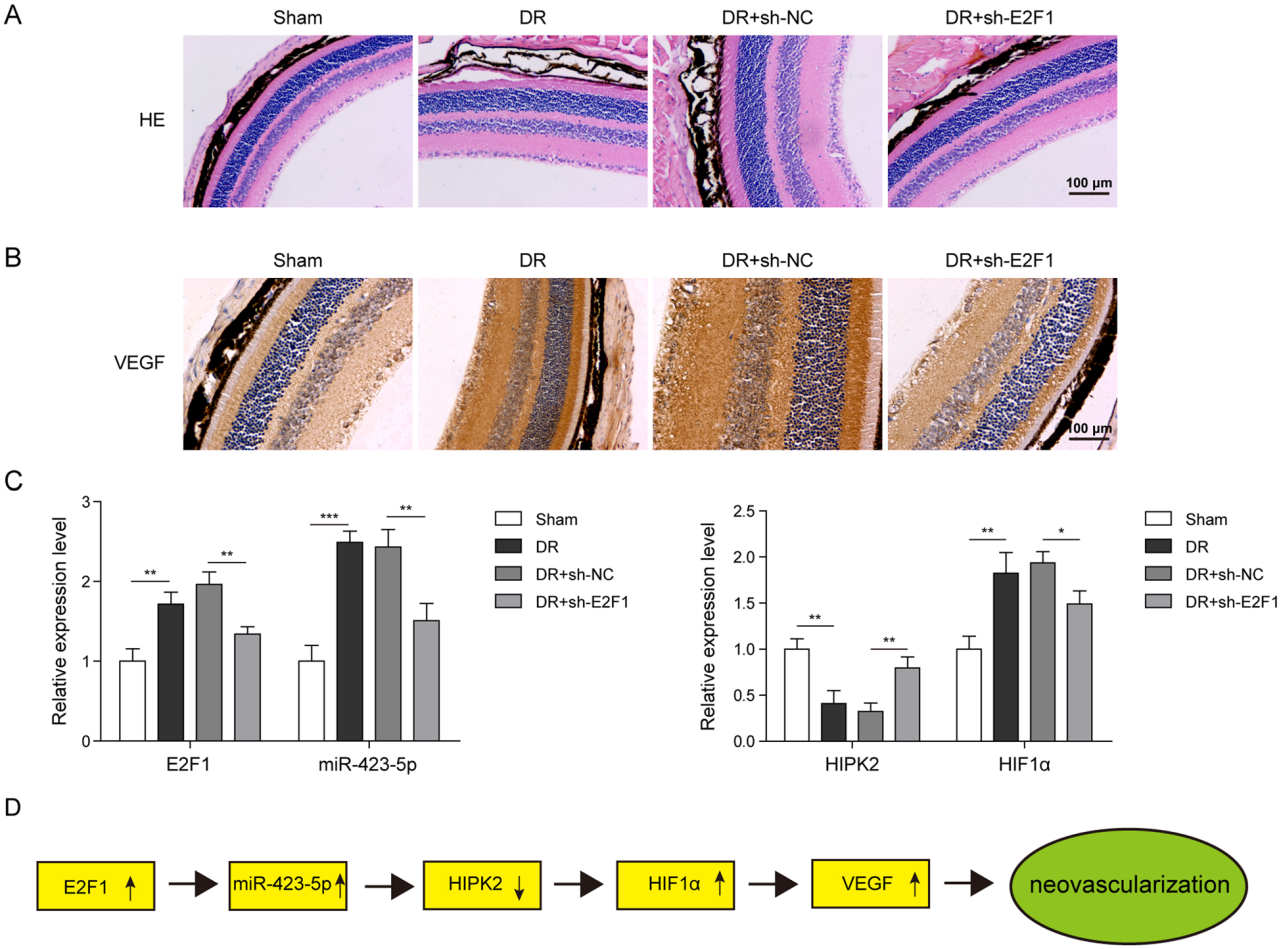
Knockdown of E2F1 suppressed angiogenesis during DR***in vivo.*** **A** DR group indicated the unclear retinal structure and the cells were arranged disorderly compared to sham group. Knockdown of E2F1 through sh-E2F1 reversed above pathological changes of retina. **B** DR group showed the increased VEGF signal in retina while sh-E2F1 suppressed DR-induced increase of VEGF level in retina compared with sh-NC. **C** DR group displayed the increased levels of E2F1 mRNA, miR-423-5p, and HIF1α mRNA but decreased HIPK2 mRNA level while sh-E2F1 reversed those changes. **D** Hyperglycemia leads to elevated E2F1, which binds to miR-423-5p promoter and activates its transcription. miR-423-5p directly targets HIPK2 and suppresses HIPK2 expression, leading to disinhibition of HIF1α/VEGF signaling, which promotes neovascularization in DR. All results were presented as the mean ± SD (n = 3). *P < 0.05, **P < 0.01, and ***P < 0.001. DR, diabetic retinopathy; E2F1, E2F transcription factor 1; HIPK2, homeodomain-interacting protein kinase 2; HIF1α, hypoxia inducible factor 1α; VEGF, vascular endothelial growth factor; NC, negative control

In the end, we evaluated the function of E2F1 in angiogenesis *in vivo*. We injected STZ to induce DR in mice. H&E staining results showed that STZ injection greatly caused the unclear retinal structure and the cells were arranged disorderly compared to sham mice (Fig. 7A). However, knockdown of E2F1 through sh-E2F1 reversed above pathological changes of retina (Fig. 7A). With IHC, we found that STZ upregulated the level of VEGF in the retina while sh-E2F1 inhibited the increase (Fig. 7B). We also measured expression levels of E2F1, miR-423-5p, HIPK2, and HIF1α. Consistent with aforementioned *in vitro* data, we found that STZ injection increased expression levels of E2F1, miR-423-5p, and HIF1α, but diminished HIPK2 mRNA level (Fig. 7C). Notably, knockdown of E2F1 reversed those changes induced by STZ (Fig. 7C). Taken together, E2F1 activates miR-423-5p transcription during DR to promote angiogenesis via suppressing HIPK2 expression to disinhibit HIF1α/VEGF signaling (Fig. 7D).

## Discussion

The growing incidence of diabetes around the world causes extensive vision loss as a result of DR, and DR is the most prevalent microvascular complication of diabetes [23, 24]. The diagnosis and management of DR remain challenging due to the complicated pathogenesis [25, 26]. Here, we explored the molecular mechanisms of neovascularization during DR by focusing on the E2F1/miR-423-5p/HIPK2/HIF1α axis. We found HG treatment of retinal endothelial cells led to upregulation of E2F1, miR-423-5p, HIF1α, and VEGF levels but a reduction in HIPK2 level. Knockdown of E2F1 or miR-423-5p repressed HG-induced enhancement on cell viability, migration, and angiogenesis. Mechanistically, we demonstrated that E2F1 activated miR-423-5p transcription during DR which directly targeted HIPK2 to promote cell viability, migration, and angiogenesis. Furthermore, we showed that knockdown of E2F1 suppressed angiogenesis during DR in vivo. Our results, for the first time, revealed the interactions between E2F1 and miR-423-5p, as well as miR-423-5p and HIPK2, and demonstrated their critical roles in DR.

E2F1 is an important transcription factor that regulates expression of many genes and it is largely involved in cell cycle regulation, cell proliferation and death [6, 27]. It lies downstream of varieties of growth factors including VEGF and can in turn activate expression of growth factor receptors such as VEGF receptor [28]. By regulating VEGF signaling, E2F1 has been implicated in angiogenesis process [29]. In addition, previous studies indicated a role of E2F1 in DR and inactivation of E2F1 suppressed HG-induced retinal neuronal death [11]. Here, we provided evidence that HG exposure upregulated the level of E2F1 in endothelial cells and knockdown of E2F1 suppressed the viability, migration and angiogenesis of endothelial cells. Together with previous research, our study demonstrated that E2F1 could participate in DR in many ways. Furthermore, we showed that miR-423-5p is the downstream effector of E2F1 and miR-423-5p mimics reversed the effects of E2F1 knockdown on viability, migration and angiogenesis. To our knowledge, this is the first report to prove that E2F1 induces upregulation of miR-423-5p. It might be interesting to examine whether this interaction exists in retinal neurons or other types of cells or conditions and that requires future studies. In addition, E2F1 has a crucial role in oxidative stress and previous studies have shown that oxidative stress is a mediator of angiogenesis [9, 29, 30]. Since hyperglycemia could induce oxidative stress, it is possible that E2F1 contributes to angiogenesis via regulating oxidative stress, which is worth further study in the future in our lab.

It is widely accepted that miRNAs play critical roles in many cellular processes and they exert their functions by suppressing the expression of their targets [31, 32]. MiR-423-5p was first identified as a biomarker of heart failure and regulated cardiomyocyte apoptosis [33]. Subsequently, many studies showed that it contributed to the development of cancers such as glioblastoma, colorectal cancer, and hepatocellular carcinoma [34–36]. Recently a study reported that miR-423-5p level was elevated in the plasma of DR patients [16], implying a potential role of miR-423-5p in DR. In the present study, we fully elucidated the function of miR-423-5p in DR and showed that miR-423-5p promoted the viability, migration and angiogenesis of retinal endothelial cells. Mechanistically, we identified HIPK2 as the crucial downstream target of miR-423-5p and knockdown of HIPK2 reversed the effects of miR-423-5p inhibitor on cell viability, migration, and angiogenesis. Previous studies have shown that miR-423-5p has multiple targets, such as β-linked N-acetylglucosamine (O-GlcNAc) transferase and trefoil factor 1 [16, 37]. Whether those targets of miR-423-5p are involved in the angiogenesis remain further explorations.

HIPK2 has been extensively characterized as a tumor suppressor in kinds of cancers [20, 38]. HIPK2 has crucial roles in controlling cell proliferation, apoptosis, and invasion, as well as angiogenesis during the hypoxic environment [39]. It has been shown that HIPK2 can suppress HIF1α/VEGF signaling to decrease angiogenesis and the degradation of HIPK2 leads to enhanced HIF1α/VEGF signaling and angiogenesis is a key mechanism of cancer progression [40]. However, whether HIPK2 is involved in DR is not well understood. Here, our results suggested that similar interaction of HIPK2/HIF1α/VEGF existed in retinal endothelial cells. Further, consistent with its role in angiogenesis during cancer development, HIPK2 acted as a suppressor of angiogenesis in DR as well by negatively regulating HIF1α/VEGF signaling. Through affecting HIPK2 expression, E2F1 and miR-423-5p regulated HIF1α/VEGF pathway to modulate angiogenesis in DR. Our study indicates that HIPK2/HIF1α/VEGF axis is a conserved signaling pathway of angiogenesis. Also, as anti-VEGF treatment approaches are explored as primary therapy for management of vision-threatening in DR, it would be interesting to look for promoters of VEGF receptors and treat the hRMCEs and hRCEs under HG with anti-VEGF drug in the future.

It cannot be denied that angiogenesis in retina only starts after many years after hyperglycemia in the clinical setting, and better control of blood sugar does not necessarily stop the process of retinal complication for long standing diabetic patients because of the metabolic memory. But, different from clinical onset, the method we used to create the DR model in mice might make the angiogenesis process more rapid. We also need to acknowledge that our research is fundamental research and there is still a gap compared to clinical research. A lot of time is needed in the future to study this mechanism in large animals and even in clinical samples.

## Conclusions

In summary, we demonstrate that E2F1/miR-423-5p/HIPK2/HIF1α/VEGF axis plays an essential role in regulating neovascularization during DR. E2F1 activates miR-423-5p transcription to promote angiogenesis of retinal endothelial cells via suppressing HIPK2 expression to disinhibit HIF1α/VEGF signaling. These results shed light on the mechanisms of DR progression and provide targets for future therapy development.

## Data Availability

All data generated or analyzed during this study are included in this article. The datasets used and/or analyzed during the current study are available from the corresponding author on reasonable request.
